# Magnesium application improves the morphology, nutrients uptake, photosynthetic traits, and quality of tobacco (*Nicotiana tabacum* L.) under cold stress

**DOI:** 10.3389/fpls.2023.1078128

**Published:** 2023-02-06

**Authors:** Jian Li, Muhammad Atif Muneer, Aihua Sun, Qinyu Guo, Yuemin Wang, Zhenrui Huang, Wenqing Li, Chaoyuan Zheng

**Affiliations:** ^1^ College of Resources and Environment/International Magnesium Institute, Fujian Agriculture and Forestry University, Fuzhou, China; ^2^ Institute of Tobacco Sciences, Fujian Provincial Tobacco Monopoly Bureau, Fuzhou, China; ^3^ Guangdong Provincial Key Laboratory of Crop Genetics and Improvement/Crops Research Institute, Guangdong Academy of Agricultural Sciences, Guangzhou, China

**Keywords:** low temperature, magnesium, growth, nutrients uptake, quality, photosynthesis, tobacco

## Abstract

Cold stress is one of the major constraints limiting the productivity of many important crops, including tobacco (*Nicotiana tabacum* L.) production and quality worldwide. However, the role of magnesium (Mg) nutrition in plants has been frequently overlooked, especially under cold stress, and Mg deficiency adversely affects plant growth and development. Here, we evaluated the influence of Mg under cold stress on tobacco morphology, nutrient uptake, photosynthetic and quality attributes. The tobacco plants were grown under different levels of cold stress, i.e., 8°C, 12°C, 16°C, including with a controlled temperature of 25°C, and evaluated their effects with Mg (+Mg) and without Mg (–Mg) application. Cold stress resulted in reduced plant growth. However, the +Mg alleviated the cold stress and significantly increased the plant biomass on an average of 17.8% for shoot fresh weight, 20.9% for root fresh weight, 15.7% for shoot dry weight, and 15.5% for root dry weight. Similarly, the nutrients uptake also increased on average for shoot-N (28.7%), root-N (22.4%), shoot-P (46.9%), root-P (7.2%), shoot-K (5.4%), root-K (28.9%), shoot-Mg (191.4%), root-Mg (187.2%) under cold stress with +Mg compared to –Mg. Mg application significantly boosted the photosynthetic activity (Pn 24.6%) and increased the chlorophyll contents (*Chl-a* (18.8%), *Chl-b* (25%), carotenoids (22.2%)) in the leaves under cold stress in comparison with –Mg treatment. Meanwhile, Mg application also improved the quality of tobacco, including starch and sucrose contents, on an average of 18.3% and 20.8%, respectively, compared to –Mg. The principal component analysis revealed that tobacco performance was optimum under +Mg treatment at 16°C. This study confirms that Mg application alleviates cold stress and substantially improves tobacco morphological indices, nutrient absorption, photosynthetic traits, and quality attributes. In short, the current findings suggest that Mg application may alleviate cold stress and improve tobacco growth and quality.

## Introduction

**Figure 1:**
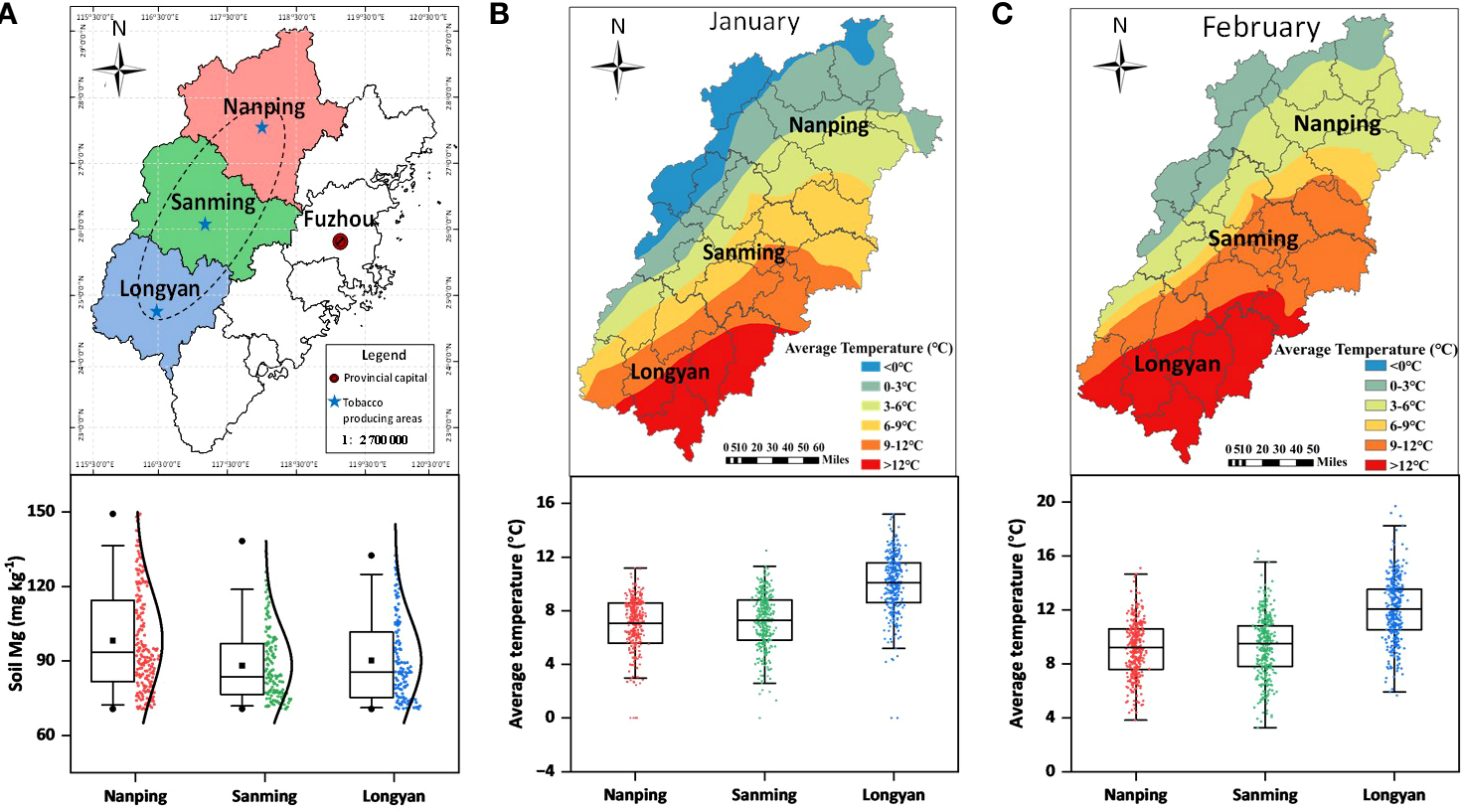
Map representing the geographic distribution of tobacco. **(A)** major flue-cured tobacco producing areas; **(B, C)** average temperature during the months of January and February.

Tobacco (*Nicotiana tabacum* L.), belonging to the family Solanaceae, originated from tropical and subtropical America but is now widely cultivated in China (Zhao et al., 2009; Zhou et al., 2020). In southeast China, which has a subtropical monsoon climate and average annual precipitation of 1558.1 mm, magnesium deficiency often occurs in acidic soils due to low cation exchange capacity and easy leaching (Guo et al., 2016). Tobacco is sensitive to cold stress. However, extreme temperatures have occurred frequently in recent years, severely restricting crops grown naturally. In the main tobacco-producing areas of Fujian Province, China, we found that the distribution of exchangeable Mg in the soil was uneven. Some areas were severely deficient in magnesium (**Figure 1A**). Meanwhile, we found that the field temperature in the early stage of tobacco seedling transplanting was lower than the temperature required for the normal growth of tobacco seedlings (**Figures 1B, C**), which profoundly affects the growth of tobacco plants and their tobacco quality.

Cold stress is a crucial environmental factor influencing the plants’ growth, yield, quality, and geographical distribution (Wang Y. et al., 2019). Tobacco (*Nicotiana tabacum* L.) is one of the most important economic crops in south China and often poses cold stress and hinders early sowing (Cui et al., 2014). Hence, tobacco growth is inhibited under cold stress and manifested by morphological traits, nutrient uptake, photosynthetic traits, and quality attributes (Li et al., 2021). For example, cold stress negatively affects the morphological traits such as shoot and root length, leaf area, stem diameter, etc., thus affecting the morphological formation of tobacco, and photosynthetic activity is also reduced and deteriorates the quality (Gechev et al., 2003; Xu et al., 2010). It has also been reported that warm-season crops are very sensitive to cold stress. For example, low temperature during the flowering time in soybean (*Glycine max*) decreases the number of pods and seeds per plant and results in low yield and poor quality (Yang et al., 2004). In cotton (*Gossypium hirsutum* L.), the cold stress for short time during later growth stage encounters the leaf spot lesions (Zhao et al., 2012). Similarly, when maize (*Zea mays*) encounters a cold injury during emergence to maturity, it results in poor germination rate and growth of leaves, inhibits the photosynthetic activity, slow filling rate and accumulation of dry matter, and finally low yield and poor quality (Prasad et al., 1994). Therefore, it is imperative to improve the cold tolerance of tobacco seedlings subjected to low temperature in the early spring.

So far, various studies have reported the adaptive responses of plants to different abiotic stresses. For instance, the application of ALA (5-aminolevulinic acid) increases the enzyme activity and nutrient content of cucumber seedlings and enhances the tolerance of cucumber seedlings to cold stress (Anwar et al., 2018). Glycine betaine and 24-epibrassinolide alleviate cold stress and improve the growth of peaches and peppers (Peng et al., 2019; Wang L. et al., 2019). The exogenous brassinolactone application effectively relieves the cold stress on the physiological metabolism of rice at the booting stage and improves yield (Wang S. et al., 2020). Salicylic acid (SA) alleviates chilling stress by improving the quality of fresh tobacco leaves, the enzyme activity of the antioxidant system, and the quality of tobacco leaves (He et al., 2020). Nevertheless, the role of Mg under cold stress to improve crop growth, yield, and quality has been neglected or not fully elucidated.

Magnesium (Mg) is an essential micronutrient for plant growth and development, as it is involved in various physiological and biochemical processes such as photosynthesis and enzymatic activities (Verbruggen and Hermans, 2013; Chen et al., 2018). Mg is considered a central atom of the chlorophyll molecule, and about 35% of total plant Mg is associated with chlorophyll and hence could play a key role in photosynthesis, energy metabolism, carbohydrate accumulation, and stress resistance (Cakmak and Kirkby, 2008; Farhat et al., 2016; Chen et al., 2018). Hence, Mg in plants directly affects yield and quality because of its diverse role in plant physiology. One of the most well-known physiological impacts of Mg shortage is its negative impact on plant photosynthetic activity and the transportation of photoassimilates to the sink organs (Geng et al., 2021). It has also been widely accepted that photosynthesis is very sensitive to cold stress, and the photosynthetic activity is significantly inhibited by the cold stress (Van Heerden and Kruger, 2000; Jin et al., 2011; Banerjee and Roychoudhury, 2019) and results in reduced synthesis and transportation of photoassimilates, and leads to poor crop yield and quality (Rude and Gruber, 2004; Wang Z. et al., 2020). Hence, understanding the effect of Mg under cold stress for improving tobacco plant growth is of great practical and theoretical significance for high yielding and quality. Therefore, in this present study, a controlled experiment was established to study the effect of Mg application under varying levels of cold stress on tobacco; i) morphological traits, ii) concentration of mineral nutrients in different plant organs, iii) photosynthetic traits and gas exchange parameters, iv) quality attributes.

## Materials and methods

### Plant culture and cold stress

The experiment was conducted under controlled conditions from March to June 2020 at the Fujian Institute of Tobacco Agricultural Sciences, Fujian, China. The seeds of the tobacco cultivar “Cuibiyihao” (*Nicotiana tabacum* L. cv. CB-1) were surface sterilized with 75% ethanol and 30% hydrogen peroxide (1:1) for 5 min. The seeds were then rinsed several times with sterilized water before being vernalized at 4°C for 12h in the dark (Forner et al., 2015; Anwar et al., 2018). In short, first, the vernalized seeds were sown in plastic trays, and seedlings were raised for 2 months under greenhouse conditions. Second, sixty healthy tobacco seedlings were selected and transplanted to hydroponic pots for adaptation to a nutrient solution environment at 25°C for 2 weeks. Third, 36 tobacco seedlings with uniform size and growth were selected and randomly divided into three equal parts and transferred to four different growth chambers, each with 12 hydroponic pots, and set the temperature of 8°C, 12°C, 16°C and 25°C respectively, because of frequent severe changes in temperature since last few years in the growing season of tobacco (**Figures 1B, C**). The black hydroponic pot with a lid, 12 cm long, 8 cm wide, and 10 cm high, with a volume of about 1000 mL, was used for the test. The black color was mainly used for shading, and the black lid had holes in the middle for the growth of plants.

The hydroponic pots of each growth chamber were divided into two sets (6 hydroponic pots for each). One set was treated with magnesium addition (+Mg) and four different temperatures, i.e., 8°C, 12°C, 16°C and 25°C, and named as +Mg8, +Mg12, +Mg16 and +Mg25, respectively. The other set without magnesium (–Mg) and the abovementioned temperatures are –Mg8, –Mg12, –Mg16 and –Mg25, respectively. It is important to investigate the effect of Mg because the main tobacco-producing areas of Fujian Province have an uneven distribution of exchangeable Mg in the soil, and some areas are severely deficient in Mg (**Figure 1A**). So, hydroponic pots of +Mg were supplemented with a modified Hoagland’s nutrient solution containing magnesium (pH 6.5-7.0; 995μM KNO_3_, 931μM Ca(NO_3_)_2_·4H_2_O, 397μM MgSO_4_·7H_2_O and 282μM KH_2_PO_4_. Micronutrients: 22.5μM Fe(II)-EDTA, 46.2μM H_3_BO_4_, 14.3μM MnCl_2_·4H_2_O, 0.76μM ZnCl_2_, 0.32μM CuCl_2_·2H_2_O, 0.13Mμ Na_2_Mo_4_·2H_2_O). While, hydroponic pots of –Mg were supplemented with modified Hoagland’s nutrient solution without magnesium (pH 6.5-7.0, 995μM KNO_3_, 931μM Ca(NO_3_)_2_·4H_2_O, 147μM Na_2_SO_4,_ and 282μM KH_2_PO_4_; micronutrients: 22.5μM Fe(II)-EDTA, 46.2μM H_3_BO_4_, 14.3μM MnCl_2_·4H_2_O, 0.76μM ZnCl_2_, 0.32μM CuCl_2_·2H_2_O, 0.13μM Na_2_Mo_4_·2H_2_O). The growth chambers were set to photosynthetically active radiation of 700 μmol·photon m^-2^·s^-1^, the relative humidity is 80%, 12 hours: 12 hours for light: dark cycle. The nutrient solution was replaced weekly. The experiment was harvested after eight weeks of temperature and Mg treatment.

### Measurement of plant morphological traits

The tobacco plants were harvested and separated into shoots and roots. Shoot and root were weighed to obtain the fresh biomass of shoot fresh weight (SFW) and root fresh weight (RFW). To determine the shoot dry weight (SDW) and root dry weight (RDW), the plant material was oven-dried for 48h at 70°C (Zhang B. et al., 2020).

### Analysis of mineral nutrients concentration in different plant organs

For different plant mineral analyses, plants’ roots and shoots were collected, oven-dried, ground, and passed through 60 mesh sieves. For nitrogen (N), phosphorous (P), and potassium (K) minerals analysis, 0.05 g of tobacco plant material of each root and shoot was digested in 5 mL of concentrated sulfuric acid (H_2_SO_4_) and 4.0 mL of hydrogen peroxide (H_2_O_2_) on digestion tubes. The digestion tubes were kept in the digestion block for approximately 2h, at a temperature of 200 ± 20°C until about 2ml of clear digestive fluid without granules was obtained. Then the deionized water was added to the digestive fluid and filtered using a 0.45-micron filter, and the extracts were stored at 4°C until nutrient determination (Huang et al., 2021). For magnesium (Mg) analysis, 0.1 g of tobacco plant material of each root and shoot was digested in a mixture of nitric acid (HNO_3_): perchloric acid (HClO_4_) in a ratio 5*v*:1*v* on the hot plate at 180°C until clear aliquot was obtained. The deionized water was added to the digestive fluid and filtered using a 0.45-micron filter, and the extracts were stored at 4°C for further analysis (Sharma et al., 2020). Total P and Mg in the samples were determined by an Inductively Coupled Plasma Optical Emission Spectrometer (ICP-OES). Total carbon (C) and total nitrogen (N) were measured by a Skalar Primacs SN100 (Netherlands, DUMAS). Total K was determined with a flame photometer.

### Determination of chlorophyll and carotenoids contents

On the day of harvest, 0.1 g of fresh leaves of tobacco seeding were taken from each replication to measure leaf total chlorophyll and carotenoid contents using an ultraviolet and visible spectrophotometer (UV-VIS). The samples were put into 25 mL 80% acetone solution. They were then kept in darkness at room temperature overnight to allow for the extraction of the leaf pigments. The mixture of chlorophyll and 80% acetone solution was examined at 663 nm, 645 nm, and 470 nm to determine chlorophyll-a, chlorophyll-b, and carotenoids, respectively. The calculation method was as follows:


$$\text{Chlorophyll}-\text{a }=\;12.7\left({\text{OD}}_{663\text{nm}}\right)\;-\;2.69\;\left({\text{OD}}_{645\text{nm}}\right)$$

$$\text{Chlorophyll}-\text{b }=\;22.9\;(\text{O}{\text{D}}_{645\text{nm}})-\;4.68\left(\text{O}{\text{D}}_{663\text{nm}}\right)$$

$$\text{Carotenoids }=\text{ O}{\text{D}}_{470\text{nm}}\times \;4$$

### Determination of leaf gas exchange and fluorescence

Leaf gas exchange parameters, including net photosynthetic rate (Pn), stomatal conductance (Gs), and intercellular CO_2_ concentration (Ci), were measured for each plant with a CIRAS-3 portable photosynthesis system (PP System, Maryland USA). Measurements were made on an upper, third fully expanded leaf of an individual plant representing each monoculture tank, typically between 09:00 and 11:00 local time, and were measured once Pn was stable after 2 to 4 min and under the following conditions: 400 μmol·mol^-1^·CO_2_, 1000 μmol·m^-2^·s^-1^ photosynthetically active radiation, 25°C leaf temperature (Lee et al., 2011; Koch et al., 2019).

For fluorescence, tobacco seedlings were kept in the dark for 30 min, and one leaf was obtained from the same rosette of each plant sample. The three to four fully expanded leaves were selected for chlorophyll fluorescence yield detection to compare the different treatment leaves. Chlorophyll fluorescence measurements were obtained using a Plant Efficiency Analyzer (PEA, Hansatech, England). The following equations were used to calculate the Fv/F_m_ (photosystemII- PSII) (Lichtenthaler et al., 2005; Liu et al., 2018);


$${\text{F}}_{v}/{\text{F}}_{m}=\;\left({\text{F}}_{m}-{\text{F}}_{0}\right)/{\text{F}}_{m}$$

$${\text{F}}_{v}=\;{\text{F}}_{m}-\;{\text{F}}_{0}$$

Where F_0_= initial fluorescence, F_v_ = variable fluorescence, F_m_ = maximum fluorescence, and F_v_/F_m_ denotes the maximal quantum yield of PSII.

### Statistical analyses

All the statistical analyses were performed using IBM-SPSS version 21.0 (IBM Corporation, New York, USA). Two-way analysis of variance (ANOVA) was performed to analyze the effect of Mg, temperature, and their interaction effect of tobacco morphological traits, minerals nutrients concentration in roots and shoots, photosynthetic traits, and quality parameters. All the measurements are presented as means with their standard errors (Mean ± SE). The statistically significant mean differences among various treatments were identified by using Duncan’s test at a significance level of *P*<0.05. The principal component analysis (PCA) was performed to evaluate the differences in biomass, minerals, nutrient content, photosynthetic traits, and quality parameters between the +Mg and –Mg under varying levels of low temperature (i.e., cold stress). The results were visualized with Origin Pro 2021 (Origin Lab Corporation, Northampton, USA).

## Results

### Effects of Mg on tobacco morphological traits under cold stress

**Figure 2:**
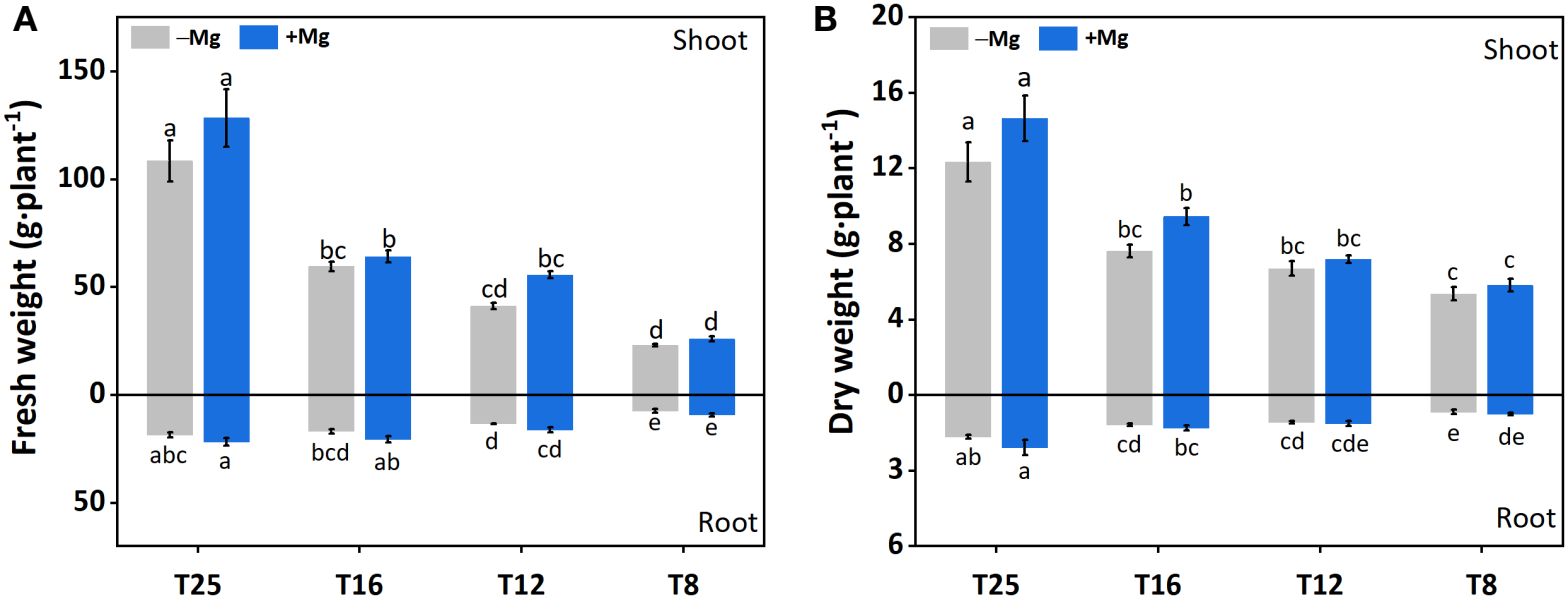
Effect of Mg application on tobacco morphological traits under different temperature. **(A)** root fresh weight (RFW) and shoot fresh weight (SFW); **(B)** root dry weight (RDW) and shoot dry weight (SDW). The different letters above the bars are indicating significant difference (Duncan *P*<0.05).

Application of Mg significantly improved the tobacco morphological traits, including shoot fresh weight (SFW), root fresh weight (RFW), Shoot dry weight (SDW), and root dry weight (RDW) as compared to –Mg under cold stress (**Figure 2**).

For SFW, the increasing trend was recorded with the increase in temperature, and a significant difference was found between different treatments. Increase in SFW under +Mg8, +Mg12, +Mg16 and +Mg25 was 12.9%, 35.0%, 7.7% and 18.2% compared to –Mg8, –Mg12, –Mg16 and –Mg25 treatments, respectively (**Figure 2A**). Similarly, RFW under +Mg treatment was significantly increased compared with –Mg treatments, while no significant difference was found at 8°C (**Figure 2A**). SDW was significantly increased under +Mg25 compared to –Mg25, while non-significant differences were recorded at 8°C and 12°C (**Figure 2B**). For RDW of the tobacco plant, significant differences were found among different treatments, and the maximum RDW was recorded at –Mg25 (1.65 g·plant^-1^) and +Mg25 (2.07 g·plant^-1^) (**Figure 2B**). The phenotypic differences for +Mg and –Mg were evident under different low temperatures. The results of two-way ANOVA showed the significant main effects of Mg and temperature and their interaction effect for RFW, while RFW showed significant effects only for Mg and temperature. In the case of SDW and RDW, Mg had no significant effect, while temperature had a significant effect (**Table 1**).

**Table 1:** Results of Two-way ANOVAs on tobacco morphological traits.

Treatment	Magnesium	Temperature	Magnesium × Temperature
Shoot fresh weight	*	*	*
Root fresh weight	*	*	ns
Shoot dry weight	*	*	*
Root dry weight	ns	*	ns

The steric symbol (*) represents the significance at P< 0.05, and ns denotes the non-significant difference.

### Effect of Mg on nutrients uptake under cold stress

**Figure 3:**
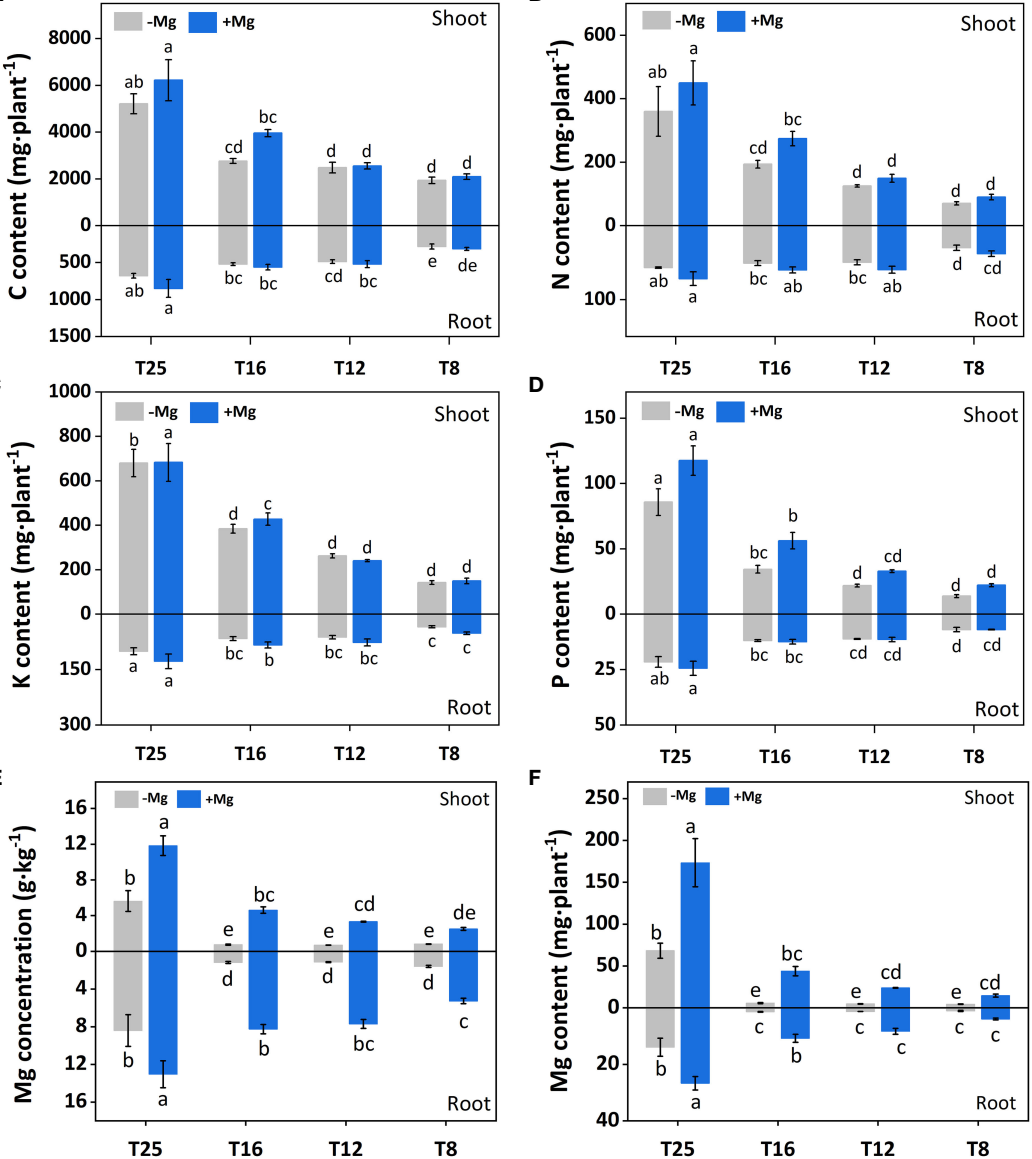
Effect of Mg application on concentration of minerals nutrients under different temperature in tobacco plant. **(A)** shoot-C content and root-C content; **(B)** shoot-N content and root-N content; **(C)** shoot-K content and root-K content; **(D)** shoot-P content and root-P content; **(E)** shoot-Mg concentration and root-Mg concentration; **(F)** shoot-Mg content and root-Mg content. The different letters above the bars are indicating significant difference (Duncan *P*<0.05).

Application of Mg under varying levels of temperature altered the nutrients uptake (**Figure 3**). For example, carbon (C) contents of the shoot were significantly higher under +Mg treatment compared with –Mg at all temperatures, and the highest C content (6221.42 mg) was recorded at +Mg25 (**Figure 3A**). The increase in shoot C content under +Mg was 8.5%, 2.9%, 43,2% and 19.3% compared with –Mg at 8°C, 12°C, 16°C, and 25°C, respectively. In contrast, root C contents were non-significant at 8°C and 12°C, while significantly higher (849.07 mg) only at 25°C (**Figure 3A**). The results of Two-way ANOVA showed that shoot and root C contents were significantly affected by the main effects of Mg, and temperature, while for interaction effect significant results found for shoot C, and non-significant for root C (**Table 2**).

**Table 2:** Results of Two-way ANOVAs on tobacco nutrients uptake.

Treatment	Magnesium	Temperature	Magnesium ×Temperature
Shoot C content	*	*	*
Root C content	ns	*	ns
Shoot N content	*	*	*
Root N content	ns	*	ns
Shoot K content	ns	*	ns
Root K content	*	*	ns
Shoot P content	*	*	ns
Root P content	ns	*	*
Shoot Mg concentration	*	*	*
Root Mg concentration	*	*	*
Shoot Mg content	*	*	*
Root Mg content	*	*	*

The steric symbol (*) represents the significance at P< 0.05, and ns denotes the non-significant difference.

Similarly, for nitrogen (N), a significant difference was observed for aboveground plant parts (i.e., shoot), and shoot N contents under +Mg treatment were significantly higher than –Mg at all temperatures and showed maximum N contents at 25°C (**Figure 3B**). For root, N contents were significantly higher at +Mg25 compared with other treatments (**Figure 3B**). Mg, temperature, and their interaction effect had a significant effect on shoot N content, while for root N, only temperature showed the significant effect (**Table 2**).

The potassium (K) contents of the shoot were not significantly affected among +Mg and –Mg treatments at 8°C, 12°C, 16°C, and 25°C temperature, while temperature had a significant effect. However, it significantly increased with increasing temperature. Maximum shoot K content (683.2 mg) was found at +Mg25 (**Figure 3C**). The root K content was significantly increased with increasing temperature and higher under +Mg application compared with –Mg under all temperatures, and maximum root K content was recorded under +Mg25 (**Figure 3C**). The Mg and temperature had significant effect on root K content. In contrast, the interaction effect for both root and shoot was non-significant (**Table 2**).

The +Mg significantly increased the shoot phosphorous (P) contents at all four temperature levels compared with –Mg. Shoot P contents significantly increased with increasing temperature, and maximum shoot contents (117.5 mg) were observed at +Mg25 (**Figure 3D**). Hence, Mg and temperature significantly affected shoot P. In contrast, Mg supplementation did not significantly affect P content in tobacco roots. While the increasing temperature also increased the root P contents, and the highest root P contents were recorded at +Mg25 (24.4 mg) (**Figure 3D**). Hence, the temperature had a significant effect on the root P content of tobacco plants (**Table 2**).

Mg treatment had a significant effect on the Mg contents of the tobacco plant. For instance, shoot Mg contents were significantly higher under +Mg treatment than –Mg under all temperature levels. The maximum shoot Mg was found under +Mg treatment at 25°C (**Figure 3E**). Similar results were recorded for root Mg contents (**Figure 3F**). Shoot and root Mg contents were maximum at +Mg25 about 173.34 mg·plant^-1^ and 26.68 mg·plant^-1^, respectively. The increase rates were 91.9% and 88.5%, respectively, compared with –Mg8. Thus, shoot and root Mg contents were significantly affected by Mg, temperature, and their interaction effect (**Table 2**).

### Effects of Mg on gas exchange parameters under cold stress

**Figure 4:**
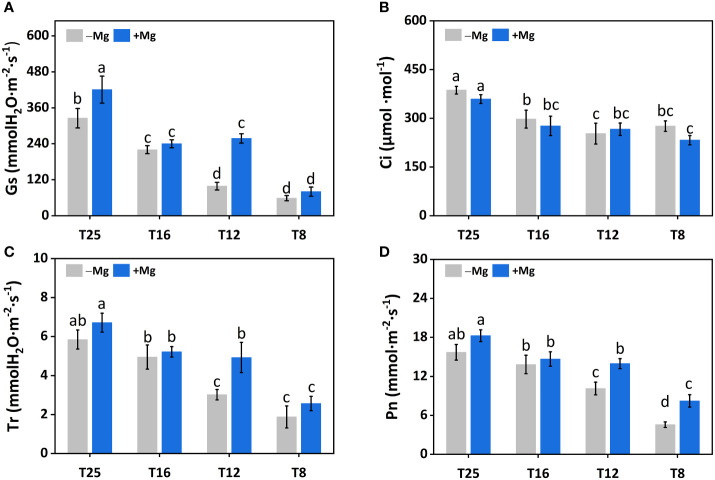
Effects of Mg application on gas exchange parameters under different temperature in tobacco plant. **(A)** Stomatal conductance (Gs); **(B)** intercellular CO_2_ (Ci); **(C)** Transpiration rate (Tr); **(D)** photosynthetic rate (Pn). The different letters above the bars are indicating significant difference (Duncan *P*<0.05).

The gas exchange parameters, including stomatal conductance (Gs), intercellular carbon dioxide concentration (Ci), transpiration rate (Tr), and photosynthetic rate (Pn), were significantly affected by the Mg application under cold stress. The Gs for both +Mg and –Mg decreased with the decrease in temperature level. The Gs under +Mg tobacco plants increased 36.9%, 62.0%, 9.0% and 29.2% compared to –Mg treatment at 8°C, 12°C, 16°C and 25°C, respectively. The highest value of Gs was recorded at 25°C, i.e., 420.67 mmol·m^-2^·s^-1^ under +Mg and 325.33 mmol·m^-2^·s^-1^ for –Mg treatment (**Figure 4A**). For Ci, there was an opposite result, the highest value was found under –Mg treatment about 359.00 μmol·mmol^-1^ at 25°C (**Figure 4B**). Whereas, the highest value of Tr and Pn was found under +Mg treatment about 6.72 mmol H_2_O·m^-2^·s^-1^ and 18.27 mmol·m^-2^·s^-1^, respectively, at 25°C (**Figures 4C, D**). Results of two-way ANOVA showed that Mg and temperature significantly affected Gs, Ci, and Pn (**Table 3**).

**Table 3:** Results of Two-way ANOVAs on tobacco gas exchange parameters.

Treatment	Magnesium	Temperature	Magnesium × Temperature
Stomatal conductance	*	*	*
Intercellular CO_2_	*	*	ns
Photosynthetic rate	*	*	*
Transpiration rate	*	*	ns

The steric symbol (*) represents the significance at P< 0.05, and ns denotes the non-significant difference.

**Figure 5:**
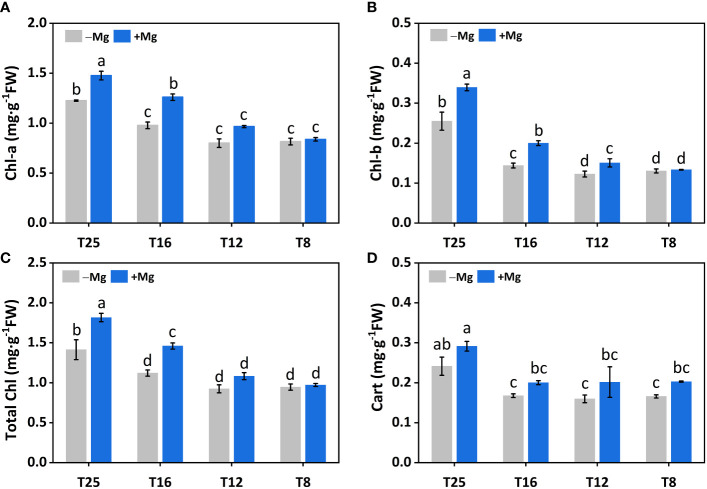
Effect of Mg application on chlorophyll of tobacco under different temperature. **(A)** chlorophyll-a (*Chl-a*); **(B)** chlorophyll-b (*Chl-b*); **(C)** total chlorophyll (Total Chl) **(D)** carotenoid (Cart). The different letters above the bars are indicating significant difference (Duncan *P*<0.05).

The Mg application had a significant effect on chlorophyll and carotenoid contents. Chlorophyll-a (*Chl-a*) content was significantly affected by Mg at 12°C, 16°C and 25°C, and maximum *Chl-a* content was recorded at +Mg25 (**Figure 5A**). A similar trend was observed for chlorophyll-b (*Chl-b*) (**Figure 5B**) and total chlorophyll contents (*Chl-a+b*) (**Figure 5C**). The carotenoid content (Cart) was increased under +Mg about 17.6%, 25.0%, 17.6% and 20.8% compared to –Mg at 8°C, 12°C, 16°C and 25°C, respectively (**Figure 5D**). Moreover, *Chl-a*, *Chl-b*, *Chl-a+b* and Cart contents were significantly affected by Mg, temperature, and their interaction effect (**Table 4**).

**Table 4:** Results of Two-way ANOVAs on tobacco chlorophyll parameters.

Treatment	Magnesium	Temperature	Magnesium × Temperature
Chlorophyll a	*	*	*
Chlorophyll b	*	*	*
Total chlorophyll	*	*	*
Carotenoid	*	*	ns

The steric symbol (*) represents the significance at P< 0.05, and ns denotes the non-significant difference.

### Effects of Mg on carbohydrate under cold stress

**Figure 6:**
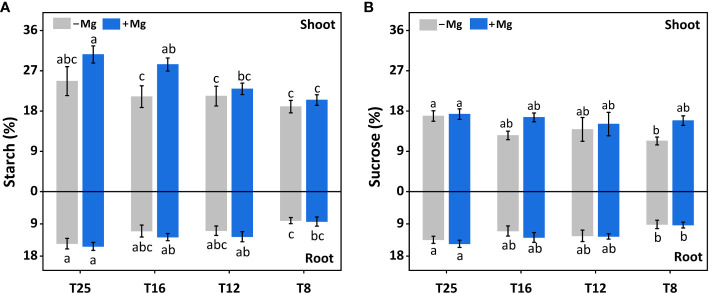
Effect of Mg application on carbohydrate of tobacco plant under different temperature. **(A)** shoot starch content and root starch content; **(B)** shoot sucrose content and root sucrose content. The different letters above the bars are indicating significant difference (Duncan *P*<0.05).

Mg supplementation had a significant effect in increasing the sucrose in tobacco plants. Similarly, the starch content increased with increasing temperature, and the maximum value was observed at 25°C 24.7% and 30.6% for both +Mg and –Mg treatment, respectively. It was increased by 7.7%, 7.5%, 33.8% and 24.1% under +Mg treatment compared to –Mg at 8°C, 12°C, 16°C and 25°C, respectively (**Figure 6A**). Hence, the sucrose contents reached a maximum about 17.3% and 16.9% at 25°C for +Mg and –Mg treatment, respectively. It was decreased by 40.2%, 8.5%, 32.1% and 2.5% under +Mg treatment compared to –Mg at 8°C, 12°C, 16°C and 25°C, respectively (**Figure 6B**). Results of two-way ANOVA have been shown that sucrose and starch were significantly affected by Mg and temperature (**Table 5**).

**Table 5:** Results of Two-way ANOVAs on tobacco carbohydrate parameters.

Treatment	Magnesium	Temperature	Magnesium × Temperature
Starch	*	*	ns
Sucrose	*	*	ns

The steric symbol (*) represents the significance at P< 0.05, and ns denotes the non-significant difference.

**Figure 7:**
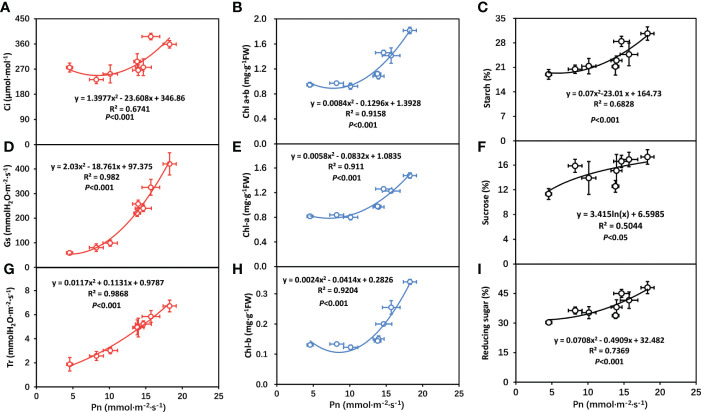
Correlation analysis of photosynthetic rate with gas exchange parameters in tobacco plants. **(A)** correlation between intercellular carbon dioxide concentration (Ci) and net photosynthetic rate (Pn); **(B)** correlation between total chlorophyll contents (*Chl-a+b*) and net photosynthetic rate (Pn); **(C)** correlation between starch and net photosynthetic rate (Pn); **(D)** correlation between stomatal conductance (Gs) and net photosynthetic rate (Pn); **(E)** correlation between chlorophyll-a contents (*Chl-a*) and net photosynthetic rate (Pn); **(F)** correlation between sucrose and net photosynthetic rate (Pn); **(G)** correlation between transpiration rate (Tr) and net photosynthetic rate (Pn); **(H)** correlation between chlorophyll-b contents (*Chl-b*) and net photosynthetic rate (Pn); **(I)** correlation between reducing sugar and net photosynthetic rate (Pn). The statistically significant mean differences among various treatments were identified by using Duncan’s test at a significance level of P<0.05.

The above results indicated that the photosynthetic system of tobacco plant had changed to some extent. With the increase of temperature, the chlorophyll of tobacco plants increased and the carbohydrate produced by the photosynthetic system also increased. In order to further clarify the relationship between chlorophyll (*Chl-a*, *Chl-b*, *Chl-a+b*), gas exchange parameters (Ci, Gs, Tr), carbohydrate (sucrose, starch reducing sugar) and photosynthetic rate (Pn), correlation analysis was conducted between chlorophyll (*Chl-a*, *Chl-b*, *Chl-a+b*), gas exchange parameters (Ci, Gs, Tr) carbohydrate (sucrose, starch reducing sugar) and photosynthetic rate (Pn). As shown in **Figure 7**, photosynthetic rate (Pn) was positively related to Ci, Gs, Tr, *Chl-a*, *Chl-b*, *Chl-a+b*, sucrose, starch and reducing, respectively.

### JIP - test

**Figure 8:**
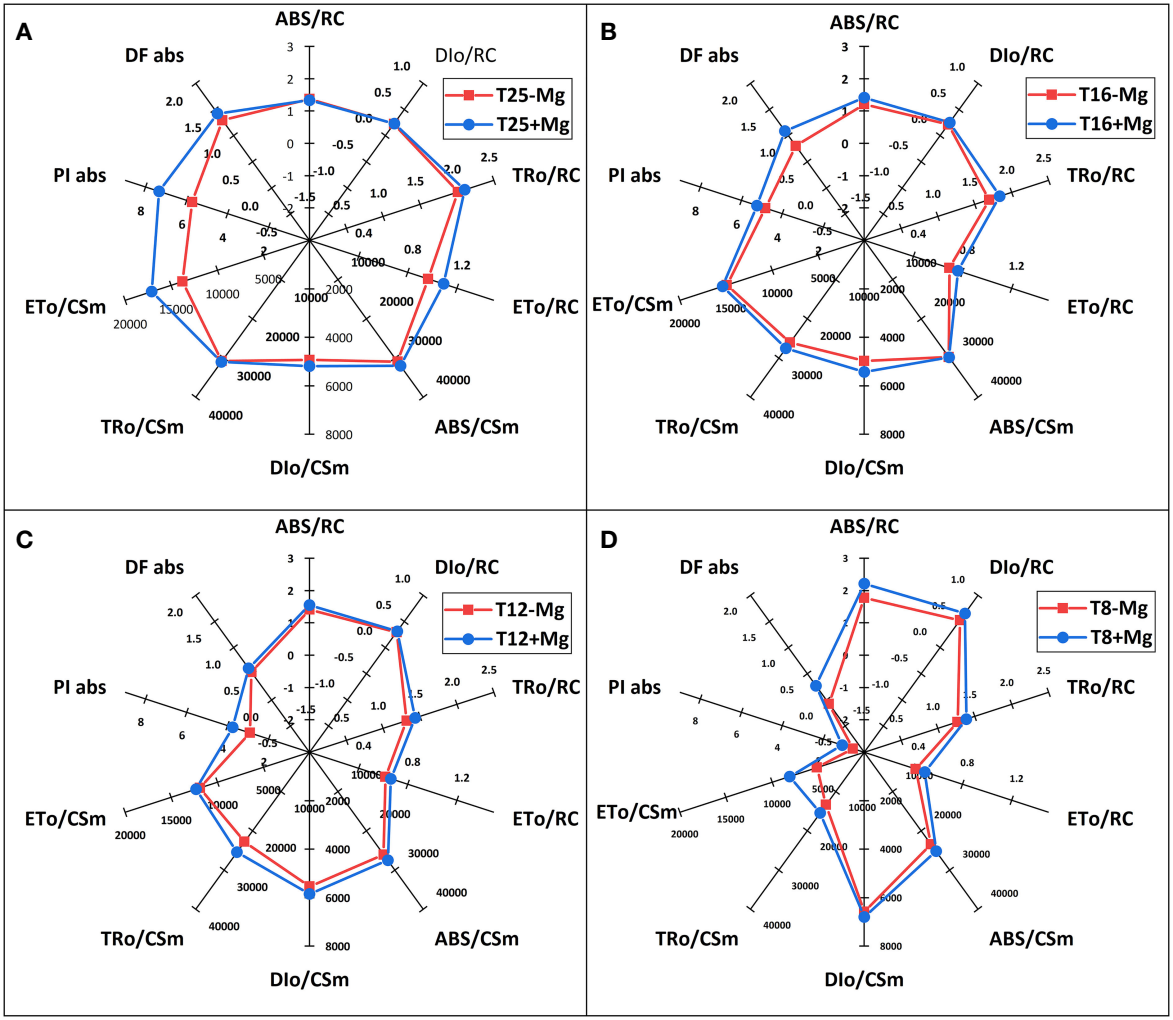
The JIP-test parameters in tobacco leaves under different temperature and Mg treatments. **(A)** 25°C; **(B)** 16°C; **(C)** 12°C; **(D)** 8°C.

The JIP-test parameters were used to analyze further tobacco’s photosynthetic behavior in various treatments (**Figure 8**). It was found that, under 25°C, the +Mg treatment did not significantly affect the photosynthetic behavior of the tobacco, but cold stress significantly affected JIP-test parameters (**Figure 8A**). With decreasing temperature, the TR_O_/RC, ET_O_/RC, ABS/CSm, TR_O_/CSm, ET_O_/CSm, PIabs, DFabs indices were significantly reduced with a decrease in temperature (**Figures 8B, C**). Cold stress significantly decreased the PI (abs), and PSII functional activity index based on standardized ABS absorption, and the lower the cold stress, the greater the decline (**Figure 8D**). Mg application significantly inhibited the decrease in PI (abs). Cold stress significantly decreased the DF (abs), and PSII driving force based on standardized ABS absorption.

Simultaneously, photosynthesis’s energy transfer in the electron transport chain was analyzed. As the results showed, after cold stress, the light energy absorbed (ABS/RC) per active reaction center (RC) and the heat dissipation (DIo/RC) were increased, suggesting that after partial RC deactivation caused by cold stress, the energy dissipation efficiency of the remaining active RC improved, which increased the burden of active RC, to resist cold environmental stress. However, +Mg treatment significantly alleviates this procedure. Additionally, cold stress decreased the excitation energy used to reduce QA (TRo/RC) and the electron transfer capacity (ETo/RC) of a single RC in tobacco, while +Mg treatment significantly alleviated the decline of ETo/RC, causing more energy to enter the electron transfer, which was conducive to maintaining the normal operation of the PSΠ linear electron transfer and increasing the tobacco’s resistance to adversity, especially cold stress.

**Figure 9:**
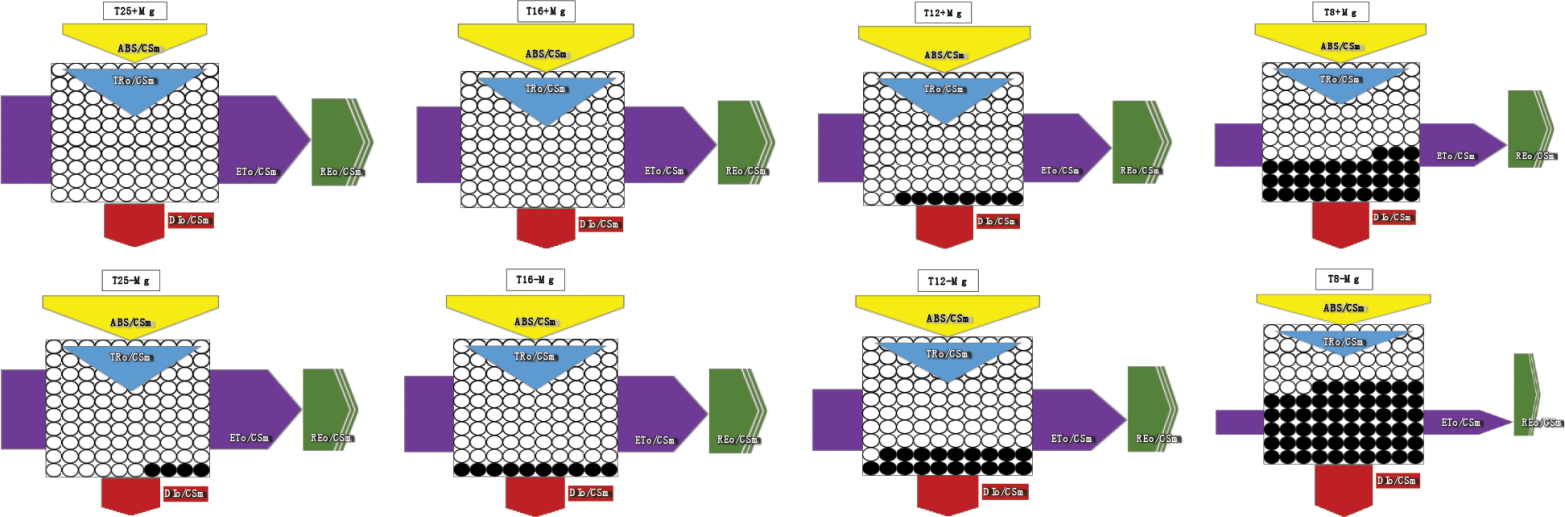
Leaf membrane model of tobacco plant with chlorophyll fluorescence parameters.

The phenomenological energy fluxes per the exciting cross sections (CS) of the grapevine leaves during the growing season are presented in **Figure 9**. 25°C measurements, the leaves were characterized by relatively high energy fluxes, which significantly decreased at 8°C. The rapid decrease of absorbed energy (ABS/RC) and electron transport per cross-section (ET/CS) was observed between 8-25°C (**Figure 9**). Then, the photosynthetic apparatus was deactivated gradually. The dissipated energy (DI/CS) did not change significantly at different temperatures but gradually increased after magnesium was added. The presented results indicate that the change of temperature (chilling stress) is the main factor of photosynthetic damage in tobacco leaves, and adding magnesium can improve the resistance to photosynthetic damage to some extent.

### Principal component analysis

**Figure 10:**
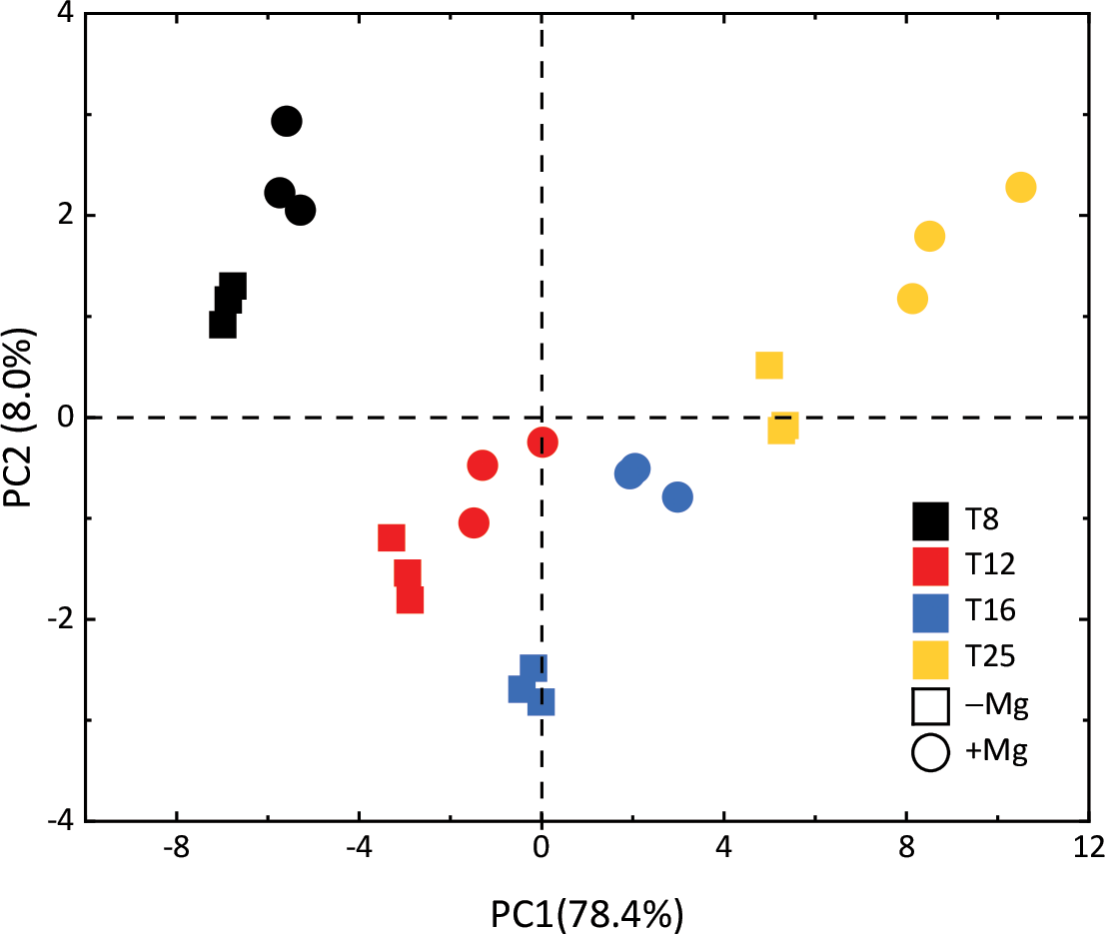
The principal component analysis (PCA) clustered the input variables of temperature and Mg into different groups.

The principal component analysis (PCA) clustered the input variables of temperature and Mg into different groups (**Figure 10**). The first two components accounted for 82.1% (68.3% for PC1 and 13.8% for PC2) of the total variation for morphological traits (SFW, RFW, SDW, RDW), nutrients contents (shoot C (S.C), root C (R.C), shoot N (S.N), root N (R.N), shoot K (S.K), root K (R.K), shoot P (S.P), root P (R.P), shoot Mg (S.Mg), root Mg (R.Mg)), photosynthetic traits (Gs, Ci, Pn, *Chl-a*, *Chl-b*, *Chl-a+b*, Cart, ABS/RC, DI_O_/RC, TR_O_/RC, ET_O_/RC, ABS/CSm, DI_O_/CSm, TR_O_/CSm, ET_O_/CSm, PIabs, DFabs), and quality attributes (sucrose and starch), and overall these showed the best performance in under +Mg at 25°C.

## Discussion

Tobacco is extremely sensitive to low temperatures, and cold stress is one of the critical abiotic stresses that significantly impact tobacco seedling growth and development (Yan et al., 2002; Zeng and Liu, 2006). Unpredictable lowering of temperature is an alarming threat to sustainable tobacco production and quality. The detrimental impacts of this low temperature can be alleviated by applying Mg, which is otherwise critical for plants to stay green under various stresses. For various reasons, the potential role of Mg under cold stress for improving tobacco growth, yield, and quality remain largely unknown. Considering the importance of Mg in improving chlorophyll synthesis and crop yield and quality (Tränkner et al., 2016), it is a matter of great interest to investigate its effects under varying levels of cold stress in tobacco.

Tobacco seedlings subjected to cold under stress under +Mg resulted in improved morphological traits in terms of SFW, RFW, and SDW (**Figure 2**). These findings are consistent with the previous findings of Mengutay et al. (2013) who found that an adequate supply of Mg significantly increased the shoot dry weight of wheat. This significant increase in biomass (i.e., SFW, RFW, SDW, and RDW) could be attributed to an increase in leaf area (Weraduwage et al., 2015) that provides higher light interception for chlorophyll synthesis (Weraduwage et al., 2015) and results in increased plant biomass. The improved root length could explain the other possible reason because Mg application significantly increases the root length that forages the soil nutrients extensively, improves the nutrient uptake, and increases plant biomass (Huang et al., 2021). The lowest plant biomass was recorded at 8°C compared to 25°C, and consistent with earlier studies that cold stress of 5°C significantly decreased the tobacco biomass (Wang Y. et al., 2019) because of the yellowing of leaves and inhibited photosynthetic activity. Hence, Mg supplementation can effectively alleviate this effect and promote the tobacco growth under cold stress, and may contribute to increase in biomass of tobacco plant.

The current study also showed the interesting results of higher root and shoot nutrients contents under +Mg treatment compared with –Mg for cold stress (**Figure 3**), suggesting the relevance of the cause-effect relationship between temperature and nutrients contents, and this effect was enhanced in the presence of Mg, and line with previous findings (Soniya et al., 2018; Dietrich et al., 2020; Perazzoli et al., 2020; Geng et al., 2021). The nutrient uptake takes place primarily through root interception (Onwuka and Mang, 2018), and the addition of Mg has a positive effect on root length, and as a result, nutrient uptake is increased (Huang et al., 2021). Moreover, it has been found that temperature and nutrient uptake had a positive relationship, and these findings were supported by the previous studies where the authors demonstrated that increasing temperature could significantly promote N, P, K, and Mg uptake (Ropokis et al., 2019; Fan et al., 2020; Vu et al., 2020). Temperature is also an important environmental factor for plant growth and development; hence, the optimum temperature may accelerate the biochemical reactions for better plant growth (Zhang et al., 2019; MacAlister et al., 2020).

Changes in photosynthetic characteristics in response to stress have received much attention in recent years. Hence, in this study, we also characterize the photosynthetic traits. We found higher chlorophyll contents, including Chl-*a*, Chl-*b*, and Cart under +Mg, and the previous finding also showed a positive correlation between chlorophyll contents and Mg (Peng et al., 2019). We found chlorophyll contents increased with increasing temperature and maximum at 25°C, suggesting a positive correlation between them, and similar results have been reported in wheat and rice (Kumar Tewari and Charan Tripathy, 1998; Zhao et al., 2020). The increase in PSII efficiency was associated with increased intracellular CO_2_ (Ci) because Mg application generally increases CO_2_ fixation, transportation, and distribution of photoassimilates that contribute to improved plant growth (Jia et al., 2021). This is consistent with our findings that gas exchange parameters, including gs, Ci, and Pn, were significantly increased under +Mg treatment and decreased under –Mg (**Figure 5**). Similar findings have been reported in broad beans (Hariadi and Shabala, 2004), indicating that Mg deficient leaves showed a decrease in Pn and vice versa. It has also been cited that Mg deficiency changes the physiological functioning of the PSII of sugar beet by decreasing the chlorophyll contents (Hermans et al., 2004). It has also been observed by other researchers that Mg deficiency results in a negative effect on photosynthesis electron transport capacity (Yang et al., 2012; Ye et al., 2019). These findings suggested that a decrease in Pn is associated with a decrease in gs and Ci.

Sucrose and starch are known as important tobacco quality indicators. It has been reported that temperature also efficiently induces sucrose and starch biosynthesis (Zhang B. et al., 2020). Plant Mg status has a pronounced impact on the transport and utilization of photosynthates, thereby significantly influencing the carbohydrate partitioning between source and sink organs (Cakmak and Kirkby, 2008). In this study, we found that sucrose and starch contents significantly improved under +Mg treatment and in line with the previous findings (Liu et al., 2008; Gerendás and Führs, 2013; Senbayram et al., 2015). It could be due to improved photosynthesis activity because various studies have elucidated that Mg has a significant role in photosynthesis by increasing chlorophyll contents (Jiang et al., 2015; Jia et al., 2021). We found that increasing temperature (25°C) increased the sugar contents compared to 8°C. The field experiments also showed a positive correlation between the temperature and sugar contents because the temperature is the control factor of enzymes in the biochemical process. Hence, with the increase in temperature, the enzymatic reaction increases, and the accumulation of carbohydrates also accelerates and results in improved sucrose and starch (Abidi et al., 2015; Sohag et al., 2020; Vosnjak et al., 2021).

The comprehensive analysis of PCA also showed the variation in morphological, the concentration of mineral nutrients, photosynthetic traits, and quality parameters for +Mg and –Mg treatments under cold stress (**Figure 9**). Similar results have also been reported that variation in morphological traits and mineral nutrients and photosynthetic traits can be attributed to the application of Mg (Ye et al., 2019; Jia et al., 2021).

## Conclusion

Mg is an essential nutrient for plant growth and development, as it is involved in various physiological and biochemical processes such as photosynthesis and enzymatic activities. However, the role of Mg in improving plant growth and development under cold stress has not been fully elucidated. This study confirmed that the addition of Mg improved the photosynthetic system and nutrient uptake of tobacco plants under cold stress. The addition of Mg increased the chlorophyll content and photosynthetic rate, and increased the carbohydrate to improve the tolerance of tobacco plants. These results confirmed that magnesium application had significant protective effect on cold stress of tobacco. Further investigation is needed in tobacco fields under various cold stress scenarios by considering the Mg application to improve the tobacco yield and quality.

## Data availability statement

The original contributions presented in the study are included in the article. Further inquiries can be directed to the corresponding authors.

## Author contributions

JL: Experimental set-up and parameters determination, data analysis, writing-original draft, software, visualization. MM: writing and reviewing. AS, QG and YW: Parameters determination, data curation and software. HZ: Writing-reviewing. WL: Investigation, conceptualization, writing-reviewing. CZ: Conceptualization, data curation, writing-reviewing and editing. All the authors contributed to the article and approved the submitted version.
