# Corrigendum: Magnesium application improves the morphology, nutrients uptake, photosynthetic traits, and quality of tobacco (*Nicotiana tabacum* L.) under cold stress

**DOI:** 10.3389/fpls.2023.1172742

**Published:** 2023-03-09

**Authors:** Jian Li, Muhammad Atif Muneer, Aihua Sun, Qinyu Guo, Yuemin Wang, Zhenrui Huang, Wenqing Li, Chaoyuan Zheng

**Affiliations:** ^1^College of Resources and Environment/International Magnesium Institute, Fujian Agriculture and Forestry University, Fuzhou, China; ^2^Institute of Tobacco Sciences, Fujian Provincial Tobacco Monopoly Bureau, Fuzhou, China; ^3^Guangdong Provincial Key Laboratory of Crop Genetics and Improvement/Crops Research Institute, Guangdong Academy of Agricultural Sciences, Guangzhou, China

**Keywords:** low temperature, magnesium, growth, nutrients uptake, quality, photosynthesis, tobacco

In the published article, there were errors in **Figures 1**, **3** as published. In **Figure 1C**, the southernmost area should be Longyan. It was mistakenly marked as Nanping. In **Figure 3F**, the column diagram of T16 for root under +Mg treatment should be filled with blue color. It was mistakenly filled with gray. The corrected **Figures 1**, **3** and their captions appear below.

**Figure 1:**
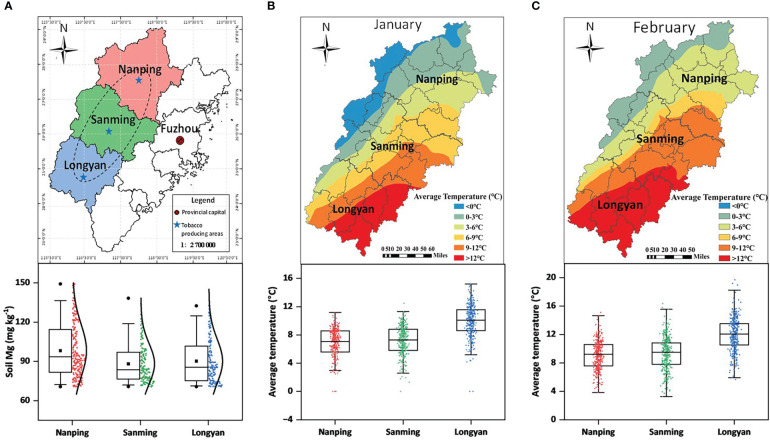
Map representing the geographic distribution of tobacco. **(A)** major flue-cured tobacco producing areas; **(B, C)** average temperature during the months of January and February.

**Figure 3:**
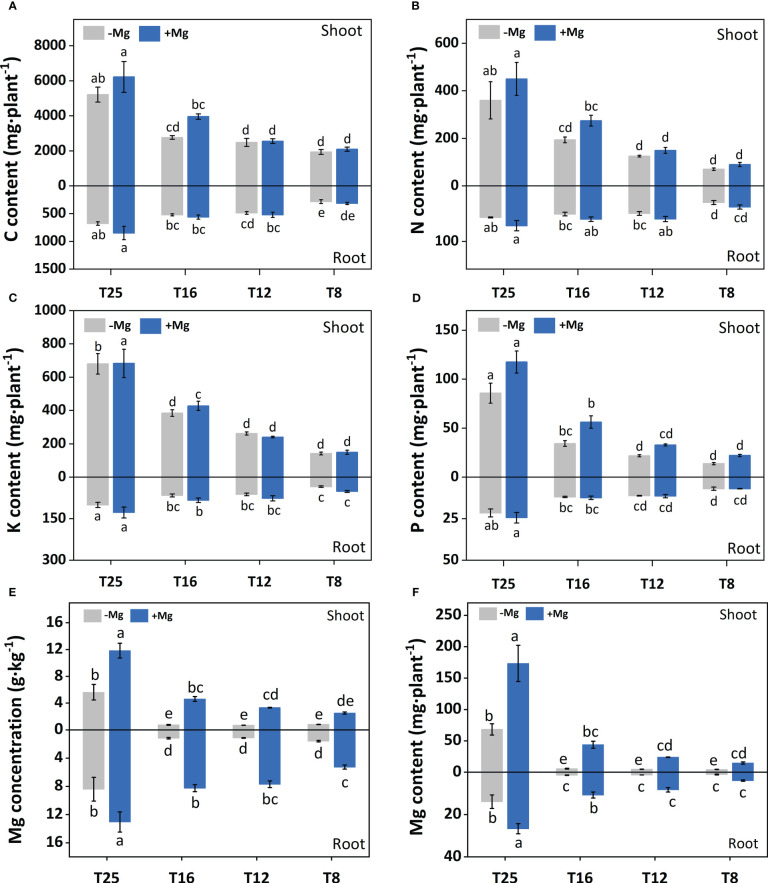
Effect of Mg application on concentration of minerals nutrients under different temperature in tobacco plant. **(A)** shoot-C content and root-C content; **(B)** shoot-N content and root-N content; **(C)** shoot-K content and root-K content; **(D)** shoot-P content and root-P content; **(E)** shoot-Mg concentration and root-Mg concentration; **(F)** shoot-Mg content and root-Mg content. The different letters above the bars are indicating significant difference (Duncan *P* < 0.05).

The authors apologize for these errors and state that this does not change the scientific conclusions of the article in any way. The original article has been updated.

